# Green synthesis of carbamates and amides via Cu@Sal-Cs catalyzed C–O and C–N oxidative coupling accelerated by microwave irradiation

**DOI:** 10.1038/s41598-021-97554-3

**Published:** 2021-09-13

**Authors:** Mahboubeh Asadi, M. Reza Naimi-Jamal, Leila Panahi

**Affiliations:** Research Laboratory of Green Organic Synthesis and Polymers, Department of Chemistry, Iran University of Science and Technology, Tehran, 16846-13114 Islamic Republic of Iran

**Keywords:** Catalysis, Organic chemistry

## Abstract

A new nano-scale Cu@salicylaldehyde-modified-chitosan (Cu@Sal-CS) was synthesized through a green, eco-friendly and cost-effective technique. The prepared catalyst was characterized using Fourier transform infrared spectroscopy (FT-IR), scanning electron microscopy (SEM), Energy-dispersive X-ray spectroscopy (EDXS), and inductively coupled plasma (ICP) analysis. The synthesized Cu@Sal-CS catalyst indicated its performance in the C–O and C–N oxidative coupling using the reaction of 1,3-dicarbonyl derivatives/2- substituted phenols with amides for the preparation of carbamates, as well as in the reaction of aldehydes and various amines in the synthesis of amides. The significant features of this work are operational simplicity of catalyst synthesis, in situ and new modification method, use of an efficient, recoverable, frequently reused and stable catalyst without any loss of catalytic activity, and high yields of the products in short times.

## Introduction

C–C and C–X couplings are among the most important reactions in organic synthesis^1^, as they can be used in the formation of new organic products^2^. Recently, transition-metal-catalyzed organic reactions through the C-H bond activation have attracted much attention from the atom- and step-economical points of view, and a variety of catalytic processes utilizing different modes for activating the available bond have been developed^3,4^. Nowadays, urethanes are considered as very important materials for the discovery and development of drugs. They are classified as one of the basic structural elements building up numerous approved human therapeutic agents^5^. Carbamates can be mainly classified into inorganic and organic categories. When the carbamate linkage is attached to any inorganic atoms, either metallic or nonmetallic, such compounds are referred to as inorganic carbamates^6^. Organic carbamates can be produced through the substitution of amino and carboxyl moieties on the unstable carbamic acid (H_2_N-COOH) with structurally diverse groups. They are considered as a stable class of compounds, whose characteristic is the linkage O–CO–NH-^6^. This group of organic compounds is widely used as agrochemicals (pesticides, herbicides, insecticides, fungicides, etc.)^7^, pharmaceuticals^8^, intermediates in organic synthesis^9^, protecting agent for amino groups in peptide chemistry^10^, and linkers in combinatorial chemistry^11,12^. Therefore, considerable efforts have been made in recent years to develop efficient and safe methodologies for carbamates synthesis^13–15^. Hofman^16^ and Curtius rearrangements^17^ are two methods applied for carbamates synthesis. For example, Ikegami et al. reported a method for the synthesis of carbamates through the use of sugar carboxylic acids, alcohols, and amines by using the Curtius rearrangement^18^. Also, gaseous carbon dioxide is another reagent used for the synthesis of carbamates through the Mitsunobu’s reaction^19^. The ordinary synthesis of carbamates involves intermediates such as chloroformates^20^ or isocyanates^21^, prepared by applying phosgene^22^ or its substitutes^1^. Phosgene is very harmful to the health of humans and the environment. Therefore, to avoid the use of toxic and harmful reagents, phosgene-free routes, including the oxidative carbonylation of amines using metal catalysts, have been widely reported^1^. Kumar and co-workers worked on the oxidative coupling of β-dicarbonyl- or 2-carbonyl-substituted phenols with *N*,*N*′-disubstituted formamides under oxidative conditions to yield carbamates by using CuBr_2_ as the catalyst and TBHP (*t*-butyl hydroperoxide) as the oxidant^1^. Azizi et al. reported that Fe_3_O_4_@EDTA-Cu(II) nanoparticles could catalyze the reaction of formamides with β-dicarbonyl compounds in the presence of TBHP as the oxidant to give enol carbamates in good yields^3^. Recently, Barve et al. reported that CuCl could catalyze the oxidative coupling of formamides with salicylaldehyde^23^. This team also reported the synthesis of carbamates in high yields utilizing a wide diversity of substrates including β-keto esters and 2-carbonyl-substituted phenol derivatives under oxidative conditions^24^. In 2017, Panahi and coworkers used nano porous metal–organic framework Cu_2_(BDC)_2_(DABCO) as a heterogeneous catalyst for the preparation of phenol carbamates^25^.

**Scheme 1:**
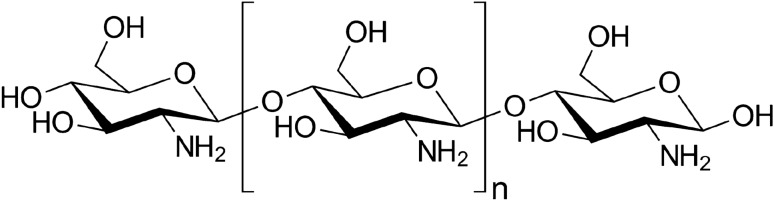
The chemical structure of chitosan.

On the other hand, an important chemical linkage in proteins and peptides structures is the amide functional group^26^. The desired approach to the formation of amide bonds (as economical and available materials) is the direct amidation of aldehydes with amines by applying a suitable catalyst. Scientists take the amide functionality into account as a key unit in polymers, organic molecules, synthetic intermediates, pharmaceuticals, and natural products. As a result, the formation of amide bonds has been of great importance among all the transformations^27^. Furthermore, several alternative approaches, including the Schmidt, Ritter, Beckmann, Ugi, Wolff, Staudinger reactions and hydration of nitriles, have proved to be efficient to enhance the fabrication of amides^28,29^. Amides play a key role in materials including natural products, synthetic intermediates, and biological and synthetic polymers, and so led to an urgent need of developing novel strategies in the chemical industry for the fabrication of amides^30^. Recently Li et al. worked on the intermolecular dehydrogenative amidation of arenes via copper (I) bromide catalyst, in which 2-pyridyl or 1-pyrazolyl was used as the chelating group and the air was employed as the terminal oxidant at 140°C^31^. In 2014, Azizi et al. reported the direct oxidative amidation of alcohols using EDTA@Cu(II) functionalized superparamagnetic nanoparticle^27^. Due to the environmental pollution resulted from the chemical industry, enormous efforts have been made toward the development of new environmentally friendly processes using heterogeneous catalysts, because of the simple catalyst removal and recovery method. The fabrication of metal nanoparticles supported on polymeric substrates is now a hot topic in the category of nanostructured materials, finding applications in numerous fields. As a biodegradable, non-toxic substrate for metal catalysts, cellulose, which is the most abundant biopolymer, has attracted innumerable global interests^32,33^. Copper (Cu) and Cu-based nanoparticles, based on the earth-abundant and cost-efficient copper metal, are recently employed in a wide range of applications, especially in the field of catalysis for the preparation of efficient catalysts^33,34^. Among the others, the composites obtained by the immobilization of transition metals on the chitosan matrix can lead to the favorable combination of the advantages of homogeneous and heterogeneous systems. Chitosan (Scheme 1) can be used as the catalyst due to the presence of amino groups on its backbone. It is abundant, renewable, and green material and simply recoverable from the reaction mixture^35^.

It has been confirmed that chitosan can adsorb transition metals via chelation^36^. One of the methods for chemical modification of chitosan to enhance its affinity as a complexing agent is Schiff base formation via the reaction of amine groups existing on the chitosan backbone with carbonyl groups^37^. Imine derivatives of chitosan have a high capacity to strongly chelate copper salts when compared with the simple chitosan^33,35^. Recently, modified chitosan biopolymer has attracted the researcher’s interest due to its many applications in catalyst^38,39^, microbial activities^37^, biosensors^40^, and etc.

In this work, modified-chitosan was obtained by treating with salicylaldehyde to enhance its copper adsorption capacity^41^. The catalyst was used to promote the copper-catalyzed C-O and C-N bonds formation via oxidative coupling. Under optimal conditions, various 2-carbonyl-substituted phenols and 1,3-dicarbonyl derivatives were utilized as substrates for the formation of carbamates (Electronic Supplementary Information (ESI), Figure S1).

## Results and discussion

### Catalyst preparation and characterization

Initially, chitosan was modified by salicylaldehyde and copper acetate through an efficient, solvent-free, and green method (ESI, Figure S2). By comparing this procedure with the previous methods reported in the literature, this protocol has some advantages such as mild and green condition, high efficiency, being straightforward, in a short time, and no need for any solvent. Here, all the steps to modify chitosan were done through mechanochemical technique, for the first time. Briefly, the Cu@modified-chitosan was synthesized in two steps. In the first step, solvent-free room temperature ball milling of the chitosan and salicylaldehyde led to the formation of an imine bond. In the second step, copper (II) acetate was added to the modified-chitosan and the mixture was grounded mechanically at room temperature. The resulting green product was characterized by using FT-IR spectroscopy, and SEM. The formation of the modified-chitosan was successfully demonstrated by the FT-IR studies^33^. The FT-IR data of chitosan, the modified-chitosan and Cu@Sal-CS are shown in Figure S3. (See ESI). By making a comparison between the IR spectra of chitosan and the modified-chitosan, the absence of –NH_2_ band and the presence of two additional bands at 1632 cm^−1^ and 1212 cm^−1^, were attributed to the formation of the imine (C=N) functional group. As it can be seen in Fig. S1, the FT-IR spectrum of the initial chitosan is identical to one reported in the literature^42^, investigating the IR-spectroscopic data of chitin and chitosan. It is evident that the presence of copper in the complex results in considerable changes in the shape of the broadband at 3000–3700 cm^−1^, attributed to various types of OH and NH vibrations in polymers. In the Cu@Sal-CS catalyst, a shoulder was observed in the spectrum of the prepared sample at 590 cm^−1^, assigned to the formation of bonds between NH_2_ groups of chitosan and Cu(I) ions^42^.

The morphology of the Cu@Sal-Cs was studied using scanning electron microscopy. The formation of the small particles at the nano-scale size was confirmed. The EDX analysis proved the presence of Cu, C, N, and O elements in the Cu@modified-chitosan structure. The results of SEM and EDX analysis are presented in Figure S4 (See ESI).

The amount of the bounded copper ions was analysed by ICP analysis. According to the results, 15.6 wt.% of the catalyst was copper, which was more than that previously reported for a Chitosan-Cu composite without chelating aid of salicylaldehyde^36^.

### Study of the catalytic activity

To explore the catalytic potential of the Cu@Sal-Cs, C‒O, and C‒N coupling reactions were investigated. Initially, C‒O coupling for the synthesis of carbamates was investigated. Accordingly, the mixture of salicylaldehyde (**1**, 1 mmol), DMF (**2**, 6 mL), and TBHP as an oxidant (1.5 eq.) in the presence of a catalytic amount of Cu@Sal-Cs (20 mg) was stirred at 80 °C in an oil bath for 1 h. After completion and separation, the carbamate **3** was achieved in a yield of 70% (Entry 2, Table 1). This result encouraged us to survey the ideal condition for the reaction by changing the reaction parameters to get the highest product yield. As shown in Table 1, the presence of both oxidant and catalyst is crucial for the reaction and in the absence of any of them, no product was observed (Entries 3 and 4, Table 1). The same reaction proceeded well (85% yield after 35 min at 80 °C) when 30 mg of the catalyst has been added to the reaction mixture (Entry 5, Table 1). Moreover, the reaction does not proceed at room temperature and requires heat (Entry 1, Table 1). Screening organic and inorganic oxidants revealed that no product was found using air, benzoyl peroxide, and hydrogen peroxide even at elevated temperatures, even at high oxidant content, and under microwave irradiation (Entries 6–9 and 11–15, Table 1). By-product was produced in the presence of benzoyl peroxide and the desired carbamate was not obtained. To investigate the importance of copper in the catalyst, some reactions were carried out by using chitosan and salicylaldehyde-modified chitosan in the same model reaction. No reaction was observed after 1 h (Entries 19–20, Table 1).

**Table 1:** Optimization of the reaction conditions of synthesis of carbamates^a^.
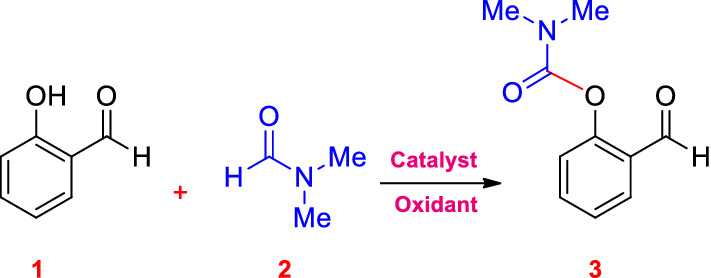

Entry	Catalyst amount (mg)	Oxidant	Oxidant amount (eq.)	Time (min)	Condition	Yield (%)^b^
1	20–30	TBHP	1.5	60	Room temperature	ND^c^
2	20	TBHP	1.5	60	Oil bath, 80 °C	70
3	–	TBHP	1.5	60	Oil bath, 80 °C	ND
4	20	–	–	60	Oil bath, 80 °C	ND
5	30	TBHP	1.5	35	Oil bath, 80 °C	85
6	30	Benzoyl peroxide	1.5–2	60	Oil bath, 80 °C	ND
7	30	Benzoyl peroxide	1.5	30	Oil bath, 100 °C	ND
8	30	H_2_O_2_	1.5–2	60	Oil bath, 80 °C	ND
9	30	H_2_O_2_	1.5–2	60	Oil bath, 100 °C	ND
10	20	TBHP	0.5–1	20	M.W. irradiation 60 °C, 200 W	60
11	20–30	Air	–	30	M.W. irradiation 60 °C, 200 W	ND
12	20	H_2_O_2_	1–2	30	M.W. irradiation 60 °C, 200 W	ND
13	20	H_2_O_2_	2	30	M.W. irradiation 80 °C, 300 W	ND
14	20–30	Benzoyl peroxide	1.5–2	30	M.W. irradiation 60 °C, 200 W	ND
15	20–30	Benzoyl peroxide	1.5–2	30	M.W. irradiation 80 °C, 300 W	ND
16	20	TBHP	1.5	15	M.W. irradiation 60 °C, 200 W	81
17	30	TBHP	1.5	12	M.W. irradiation 60 °C, 200 W	90
18	30	TBHP	1.5	4	M.W. irradiation 80 °C, 300 W	94
19	30^d^	TBHP	1.5	60	M.W. irradiation 60 °C, 200 W	ND
20	30^e^	TBHP	1.5	60	M.W. irradiation 60 °C, 200 W	ND

^a^Reaction condition: salicylaldehyde (1 mmol), Cu@Sal-Cs as a catalyst, DMF (6 mL)

^b^All yields were determined by GC.

^c^Product was not detected

^d^Chitosan was as a catalyst

^e^Salicylaldehyde-chitosan was as a catalyst.

The model reaction has also been studied under microwave irradiation. As it is clear from Table 1, microwave irradiation at 200 W/60 °C affords the product in a shorter reaction time (15 min) with an 81% yield (Entry 16). Increasing the amount of the catalyst to 30 mg affects the yield considerably (Compare entries 16 and 17, Table 1). Also, increasing the power and temperature resulted in higher yield and increased the reaction speed, and reduced the reaction time (Entry 18, Table 1). According to the results, we decided to use microwave irradiation for the coupling reaction. In conclusion, 30 mg of Cu@Sal-Cs catalyst and 1 mmol of the 2-carbonyl-substituted phenols with amides (6 mL) in the presence of 1.5 eq. of TBHP, under microwave irradiation was chosen as the optimized reaction condition. The model reaction has been tested in gram scale. Briefly, 300 mg of the catalyst was added to the mixture of DMF (60 mL) and salicylaldehyde (1.2 g, 10 mmol) in the presence of TBHP (15 eq.) as oxidant. The reaction mixture was subjected to microwave irradiation (80 °C /300 W, 10 min). The product (2-formylphenyl dimethylcarbamate) was synthesized in 94% yield (1.82 g).

After optimizing the reaction conditions, the generalization capability of this method was shown using the 2-carbonyl-substituted phenols and amides to construct a library of phenol and enol carbamates as shown in Table 2^1,3,23,25,43^. We also applied this methodology in the synthesis of β-ketocarbamates using 1,3-dicarbonyls and different formamides (Entries 20–24, Table 2). The importance of this type of reaction is that the aldehyde remains intact in the presence of the oxidant. We have also investigated the effect of the carbonyl group on *ortho* substitutions of phenols, demonstrating that the substrates possessing no *ortho* substituent do not react (Entry 26, Table 2).

**Table 2:**
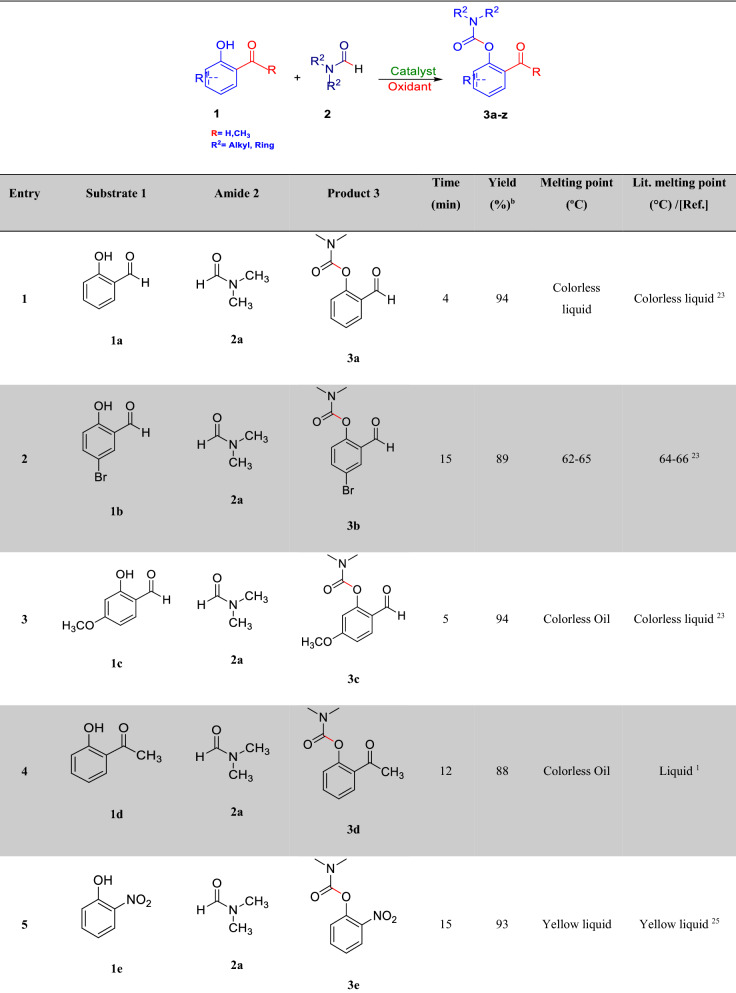

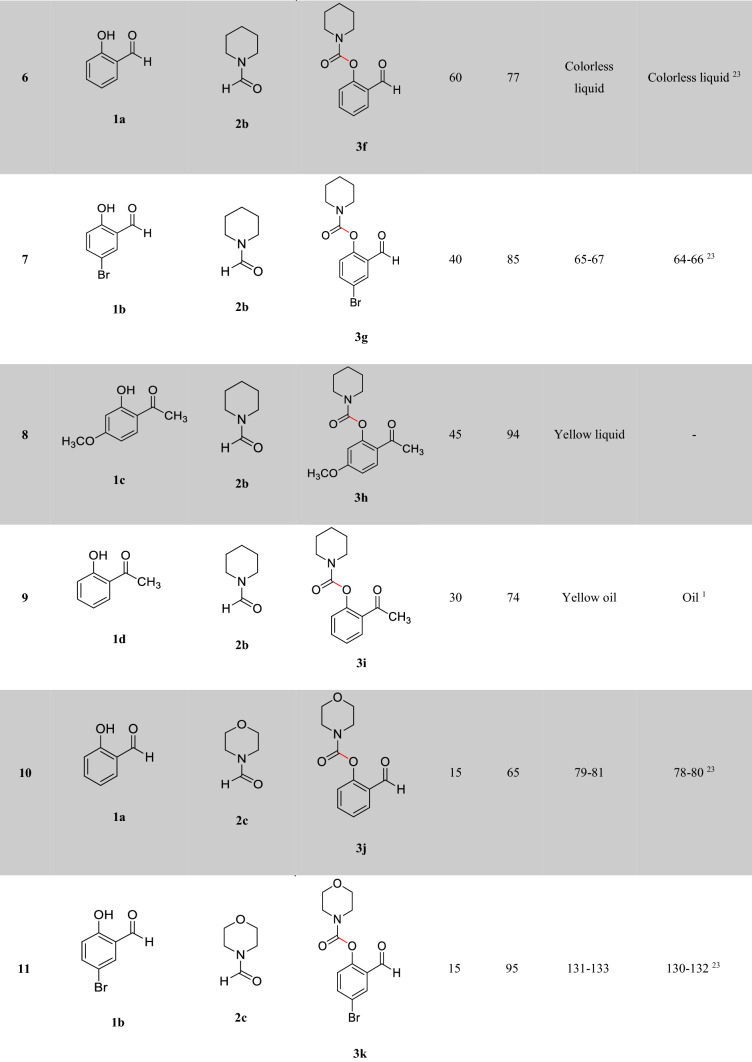

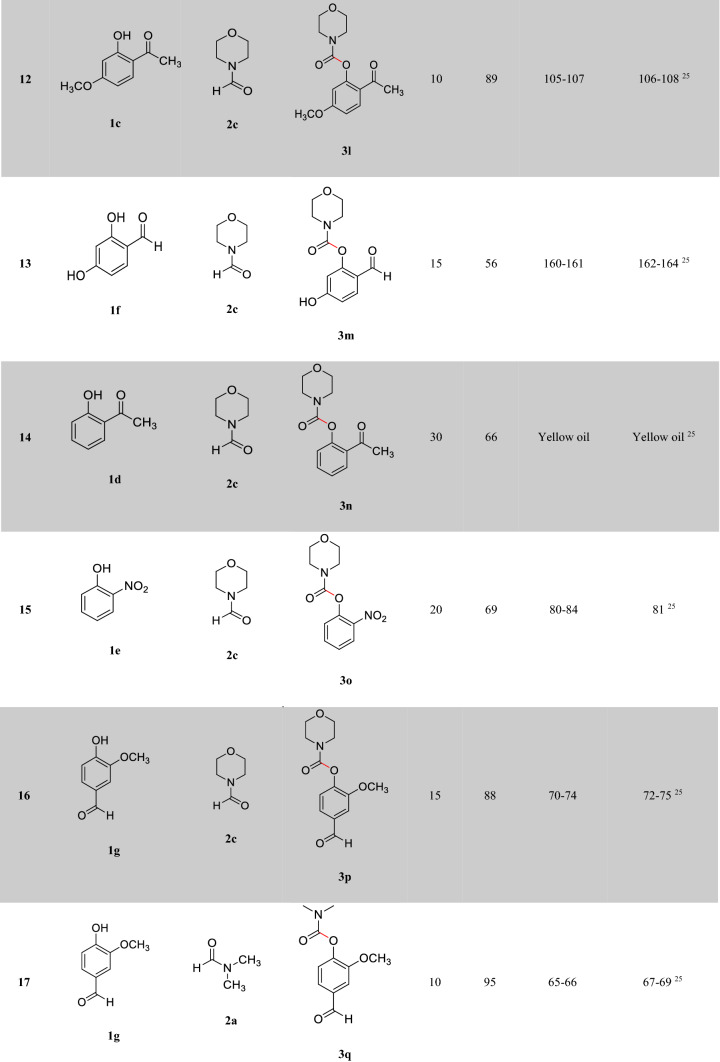

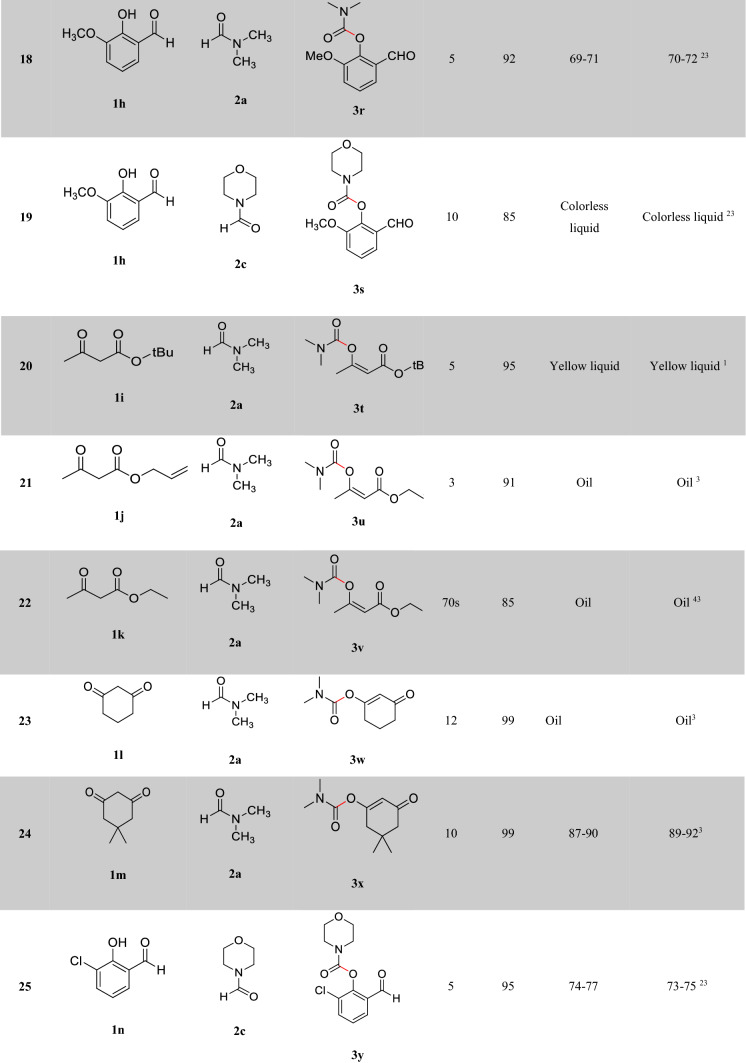

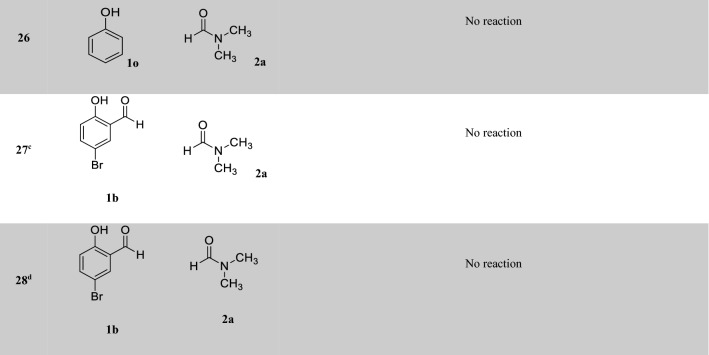
Oxidative C–O coupling for the synthesis of carbamates catalyzed by Cu@Sal-CS^a^.

^a^Substituted phenols (1 mmol), amide (6 mL), Cu@Sal-Cs catalyst (0.03 g), TBHP (1.5 eq.), M.W irradiation (300 W)

^b^All yields were determined by GC

^c^[2,2,6,6-tetramethylpiperidine-1-oxyl as radical scavenger (TEMPO) used as an oxidant

^d^Hydroquinone as an oxidant

### Possible mechanism

A plausible mechanism has been proposed for the reaction catalyzed by Cu@Sal-CS (ESI, Figure S5). In the proposed mechanism, at first, a complex is formed between the substrate (**1**) and the catalyst (**I**). Microwave irradiation decomposes TBHP (**2**) to form hydroxyl and tert-butoxyl (**3**) radicals, which in turn transform formamide to the radical **6**. The complex **III** will be formed between the radical **6** and complex **II**. Finally, this complex affords the desired carbamate. Similar to the 2-carbonyl-substituted phenol complex, a coordinated complex is suggested for the enol tautomer of the dicarbonyl moiety. This complex can be transformed to different carbamates. To demonstrate that this reaction absolutely promotes via a radical mechanism, we used the radical scavengers 2,2,6,6-tetramethylpiperidine-1-oxyl (TEMPO) and hydroquinone, and no reaction was observed in these cases (Entries 27, 28, Table 2).

### C–N coupling of benzaldehyde derivatives with amines to amides

The successful C-N coupling of benzaldehyde derivatives with amines was found to be facilitated in high yields in the presence of the Cu@Sal-Cs catalyst with TBHP and resulted in the desired amide products (**3**) (ESI, Figure S6). At first, to select the best and optimum condition, an amount of 20 mg catalyst was tested with 1 mmol *p*-methoxybenzaldehyde and 6 mL of pyrrolidine at room temperature. After 60 min no reaction occurred (Entry 1, Table 3). In the next step, 1.5 eq. TBHP as oxidant was added, but no amide was obtained (Entry 2, Table 3). Increasing the temperature to 80 °C resulted in 70% yield (Entry 3, Table 3). The use of 30 mg catalyst in the presence of 1.5 eq. TBHP led to an increase of yield in lesser reaction time (Entry 4, Table 3). Additionally, the catalytic activity of 30 mg catalyst in the presence of ethanol and acetonitrile, as the solvent, and without the oxidant was tested, which did not result in the formation of the desired product (Entries 5–6, Table 3). By the addition of TBHP, this reaction represented good yields. The reaction has also been studied under microwave irradiation. It was found that using a catalyst (30 mg), oxidant TBHP (1.5 eq), in the absence of solvent, and at the power of 300 W, led to the formation of the amide in good yield (Entry 9, Table 3).

**Table 3:** Optimization of the reaction conditions of amide synthesis.
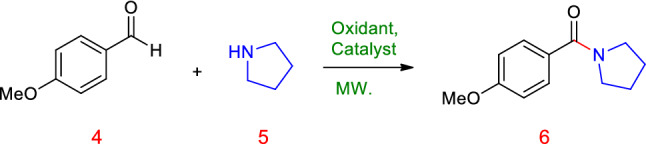

Entry	Catalyst amount (g)	Oxidant	Oxidant amount (eq.)	Solvent	Time (min)	Condition	Yield (%)
1	0.02	–	–	–	60	Room temperature	–
2	0.02	TBHP	1.5	–	60	Room temperature	–
3	0.02	TBHP	1.5	–	60	Oil bath, 80 °C	70
4	0.03	TBHP	1.5	–	60	Oil bath, 80 °C	78
5	0.03	–	–	Ethanol	60	Oil bath, 80 °C	–
6	0.03	–	–	Acetonitril	60	Oil bath, 80 °C	–
7	0.03	TBHP	1.5	Acetonitril	60	Oil bath, 80 °C	85
8	0.03	TBHP	1.5	Ethanol	60	Oil bath, 80 °C	82
9	0.03	TBHP	1.5	–	30	M.W. irradiation, 80 °C, 300 W	81

With the optimized conditions in hand, we further investigated the scope of the reaction using different substituted benzaldehydes and amines as shown in Table 4^26,44–47^. A variety of aromatic aldehydes having electron-donating or electron-withdrawing substituents, regardless of their positions, participated well in the reaction, indicating no obvious electronic impact. Substituents such as OMe, F, Cl groups on the phenyl ring (Entries 1, 4, 9, 14, Table 4)^26,45,46^ were also found to be compatible under standard reaction conditions and did not hamper the reaction processes. Aldehydes bearing several aliphatic groups such as –CH_3_ afforded the corresponding product in good yields, as well (Entries 7,8, Table 4)^26,45^. It is noteworthy that not only secondary amines but primary amines reacted well.

**Table 4:**
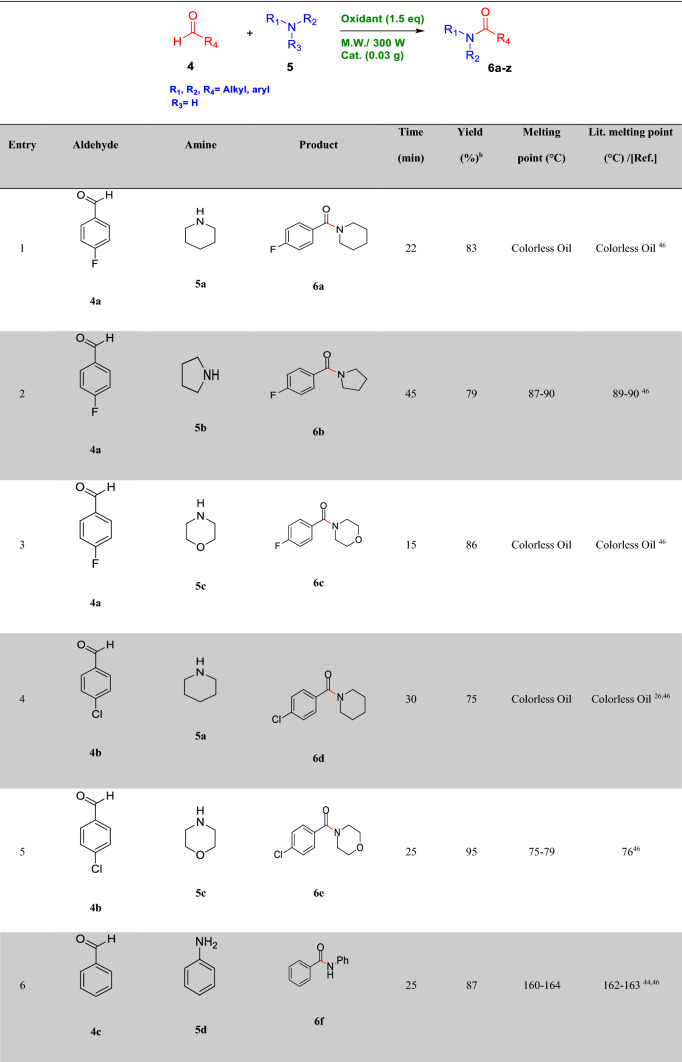

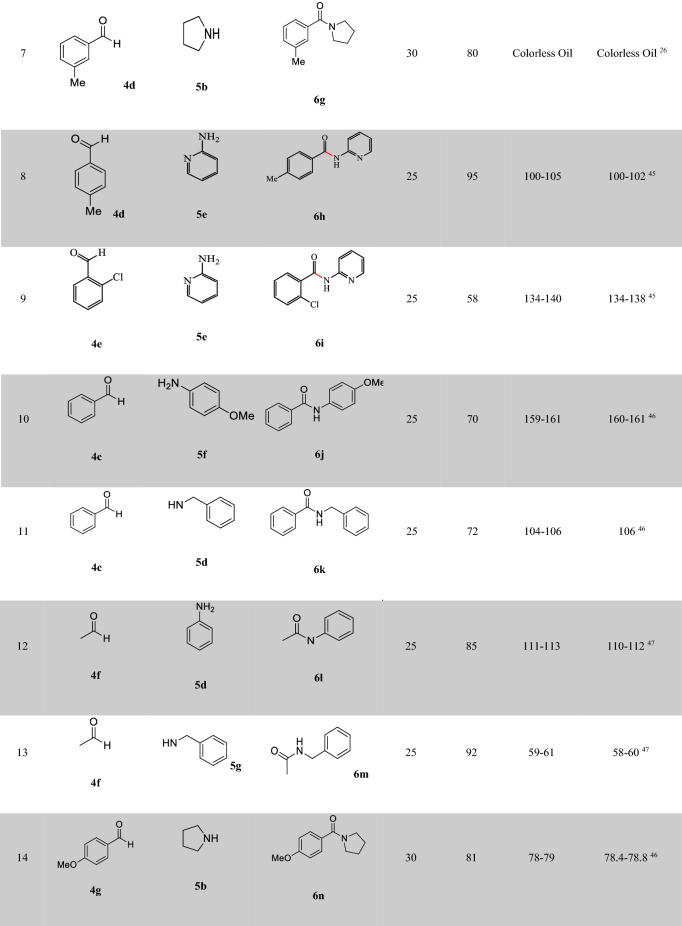
Oxidative C − N coupling for synthesis of amides catalyzed by Cu@Sal-CS^a^.

^a^Aldehyde (1 mmol), amine (6 mL), Cu@Sal-Cs catalyst (0.03 g), TBHP (1.5 eq.), M.W irradiation (300 W)

^b^All yields were determined by GC

On the other hand, to demonstrate the advantages of our catalyst over other catalysts reported in literature for the synthesis of the carbamates and amides, we compared the yield and condition of the reaction with other catalysts, based on the amount of the catalyst, the amount of oxidant and reaction time (Table 5). The present catalyst has the advantages of being green, recoverable, and affords the products in short reaction times and with high yields (Entry 4, Table 5).

**Table 5:** Comparison of the catalytic activity of Cu@Sal-CS with other similar catalysts reported in the literature for preparation of carbamates.

Entry	Reaction conditions	Product	Yield (%)	Reference
**1**	CuCl (1–2 mol %)/TBHP (6.0 eq.)/80 °C/15 min	**3a**	96	^23^
**2**	Cu_2_(BDC)_2_DABCO synthesized by ball mill (20 mg)/TBHP (1.5 eq.) 80 °C/30 min	**3a**	99	^25^
**3**	Cu-quinoline-2-carboxaldehyde@amine-SiO_2_@Fe_3_O_4_ (30 mg)/TBHP (6 eq.) /70 °C /12 min	**3a**	99	^48^
**4**	Cu@modified-chitosan (30 mg)/TBHP (1.5 eq.)/300 Watt, M.W. irradiation, 80 °C/4 min	**3a**	94	In this study

The result of comparison between catalytic activity of Cu@modified-chitosan and other catalysts in literature in the reaction between aldehyde **1d** and amide **2c** forming **3n** is reported in Table 6^26,46,49^. It can be clearly seen that our catalyst is superior to other catalysts in terms of time, higher yield, being green, and availability (Entries 1–3, Table 6).

**Table 6:** Comparison of the catalytic activity of Cu@Sal-CS with other similar catalysts reported in the literature for preparation of amids.

Entry	Reaction condition	Product	Yield (%)	Reference
**1**	Phenazinium salt (1–2 mol%)/Air, 24 Watt household lamp, THF/ambient temp/20 h	**3n**	72	^26^
**2**	[Li(DME)_3_][YbL_2_] (2 mol%)/THF/25 °C/3 h	**3n**	56	^46^
**3**	Photocatalyst (1 mol%)/CFL,O_2_ (balloon) THF(2 mL)/20 h	**3n**	16	^49^
**4**	Cu@modified-chitosan (0.03 g)/TBHP (1.5 eq.)/300 Watt/30 min	**3n**	81	In this study

### Reusability of the catalyst

The reusability of Cu@modified-chitosan in the synthesis of carbamates and amides was also studied. After each reaction, the catalyst was easily filtered and washed with ethanol to remove any organic impurities. It was then dried at 80 °C and used for the next cycle. As shown in Figure S7 (See ESI file), the catalyst could be reused in five successive reactions without any significant loss of its catalytic activity. The SEM image and FT-IR spectra of the recovered catalyst after the fifth run demonstrated that its structure remained intact (ESI, Figure S8).

In order to evaluate the chelation strength, ICP analysis for the recycled catalyst from the last run of the model reaction was compared to the fresh catalyst. According to the results, copper content of the recycled catalyst after the fifth run was measured as 15.2%, which was slightly less than in the fresh catalyst (15.6%). Taking into account that only 0.4% of copper ions was lost, this can suggest an acceptable enough strong chelation of copper ions by the substrate.

## Conclusions

To draw the conclusion, we efficiently synthesized Cu@Sal-Cs catalyst by using the solvent-free ball milling technique. This catalyst was utilized in the C-O and C-N bond formation for the synthesis of carbamates and amides with excellent yields and selectivity. The advantages of this study are the introduction of a green, selective, efficient, and easily recoverable catalyst in the oxidative coupling reactions. This catalyst was easily recovered without loss of its catalytic activity and selectivity. The advantages of our method are the easy separation and reusability of the fabricated catalyst. Moreover, during the reaction, the oxidation-sensitive functional groups (as formyl groups) remain intact. The use of microwave irradiation in the reaction process facilitates the reaction.

## Experimental section

### Materials and instrumentation

All the reagents and starting materials used in this research were of analytical grade, obtained commercially from Merck or Sigma–Aldrich and used without further purification. The chitosan used was with high molecular weight 600,000 to 800,000 Da. A MM400 Retsch, ball-milling apparatus was used for the solvent-free synthesis reactions. Two stainless steel balls with a diameter of 7 mm were used in a 10 mL stainless steel container at a frequency of 28 Hz. An industrial microwave Milestone MicroSYNTH was used for accelerating of reactions. The progress of the reactions was monitored by TLC (Thin Layer Chromatograghy) or GC (Gas Chromatography) and the actual loading of copper was determined by Inductively Coupled Plasma (ICP) analysis on sequential plasma spectrometer, Shimadzu (ICPS-7000 ver. 2). FT-IR spectra of products were recorded with a Shimadzu 8400s FT-IR spectrometer using potassium bromide pellets. ^1^H NMR and ^13^C NMR spectra were recorded with a Bruker DRX-500 Avance spectrometers with Me_4_Si as the internal standard.

Step by step modification of chitosan biopolymer with salicylaldehyde and copper (II) acetate:

### Synthesis of salicylaldehyde-chitosan (modified-chitosan)

The catalyst was synthesized by a similar procedure reported in literature^33^. Using the ball mill technique and solvent-free process without heating in a short time is the innovation in this study. A mixture of chitosan (2 g) and salicylaldehyde (0.87 mL) was ball milled at 28 Hz, under the solvent-free condition at room temperature. The process was monitored by TLC and completed within 40 min. Then obtained crude was washed with hot ethanol (3*10 mL) and dried at 60 °C to yield modified-chitosan as a yellow powder (Sal-Cs).

### Synthesis of Cu@Sal-CS

1 g of the modified-chitosan obtained from the previous step and Cu(OAC)_2_.H_2_O (1.5 g) were mixed by using the ball mill for 80 min at room temperature. After the completion of the reaction, the mixture was filtered, washed several times with hot ethanol, and then dried at ambient temperature, to produce the green powder of Cu@Sal-CS (ESI, Figure S9).

### One-pot modification of chitosan biopolymer by using salicylaldehyde and copper (II) acetate

In a 10 mL stainless steel ball mill container equipped with two 7 mm stainless steel balls, 2 g of chitosan with 0.87 mL of salicylaldehyde was placed and mixed at a frequency of 28 Hz for 10 min. After that, 1.5 g of Cu(OAC)_2_ was added to this mixture and milled at the same frequency for 1 h at room temperature to complete. The resulting solid powder was washed several times with hot ethanol, then dried at ambient temperature to yield Cu@Sal-CS as a green powder.

### General procedure for synthesis of carbamates through the C–O coupling reaction

A 20-mL round-bottomed flask was charged with 2-substituted phenols or 1–3 dicarbonyl derivative (1 mmol), formamide derivatives (6 mL), Cu@Sal-CS (30 mg), and t-butyl hydroperoxide (TBHP, 70 wt.% in water, 1.5 eq.) as oxidant. The reaction mixture was irradiated under microwave condition for the desired time at 80 °C with the power of 300 Watt. The reaction progress was monitored by TLC or GC techniques. After the completion of the reaction, the catalyst was separated by filtration and the obtained solution was extracted with ethyl acetate by decanter funnel, and washed with water (3*5 mL). The solvent was removed in vacuum and the crude product was purified by plate chromatography or column chromatography on silica gel (Ethyl acetate/*n*-hexane: 1/9) to afford the pure product.

All of the products were identified by comparison of their melting point and/or spectral data with those reported in previous literature.

### General procedure for the synthesis of amides through the C–N coupling reaction

Similar to the oxidative coupling reaction of C-O for the synthesis of carbamates, 30 mg of the catalyst was added to the mixture of amines (1.3 mmol) and aldehyde derivatives (1 mmol) in the presence of TBHP (70 wt.% in water, 1.5 eq.) as oxidant agent. The reaction mixture was subjected to microwave irradiation (80 °C /300 W). The progress of the reaction was monitored by TLC or GC analysis. After the reaction was complete, followed by extraction and purification to yield desired amides. The catalyst was separated by filtration and the reaction mixture was extracted from aqueous mixture with ethyl acetate by decanter funnel, and washed with water (3*5 mL). The solvent was removed in vacuum and the crude product was purified by plate chromatography or column chromatography on silica gel (Ethyl acetate/*n*-hexane: 1:5) to afford the pure amide. In the case of solid products, recrystallization from ethanol was chosen.

## Supplementary Information

Supplementary Information.
