# Supplementary material for: Green synthesis of carbamates and amides via Cu@Sal-Cs catalyzed C–O and C–N oxidative coupling accelerated by microwave irradiation
Source: Sci Rep. 2021 Sep 13;11:18105. doi: 10.1038/s41598-021-97554-3 (PMC8437951; doi:10.1038/s41598-021-97554-3)
Supplement: Supplementary file 1 — Supplementary Information. [file 41598_2021_97554_MOESM1_ESM.docx]

**Green Synthesis of Carbamates and Amides via Cu@Sal-Cs Catalyzed C-O & C-N Oxidative Coupling Accelerated by Microwave Irradiation**

**Mahboubeh Asadi, M. Reza Naimi-Jamal*, Leila Panahi**

*Research Laboratory of Green Organic Synthesis & Polymers, Department of Chemistry, Iran University of Science and Technology, 16846-13114 Tehran, I. R. Iran.* *Tel. /Fax: (+) 9821-77240289*

**E-mail:* [*naimi@iust.ac.ir*](mailto:naimi@iust.ac.ir)

| **Content** | **Page** |
| --- | --- |
| Title page | **S1** |
| **Figure S1.** Synthesis of enol and phenol carbamates by oxidative coupling. | **S2** |
| **Figure S2**. Preparation procedure of Cu@Sal-CS | **S3** |
| **Figure S3.** FT-IR spectra: **a)** Chitosan, **b)** Salicylaldehyde-chitosan, and **c)** The Cu@modified-chitosan sample. | **S4** |
| **Figure S4**. Analysis of Cu@modified-chitosan: **a**) SEM image, **b**) The EDX diagram. | **S5** |
| **Figure S5.** Proposed mechanism for Cu@Sal-CS catalyzed synthesis of carbamates. | **S6** |
| **Figure S6.** Synthesis of amides by oxidative coupling.  **Figure S7.** Reusability of the catalyst in the model coupling reactions at the optimized condition. | **S7**  **S8** |
| **Figure S8.** Left: FT-IR spectra: Recycled catalyst, right: Scanning electron micrograph of the recycled catalyst | **S9** |
| **Figure S9.**.Schematic structure of the synthesized Cu@Sal-CS | **S10** |
| ^1^H NMR, ^13^C NMR and IR spectral data for selected compounds (**3h**, **3i**, **6l**, **6m**) and IR spectral data for some compounds  IR spectral data for some compounds  **Figure S10.** ^1^H NMR: 3-Methoxy-Piperidine-7-carboxylic acid 2-acetyl-phenyl ester **(3h)**  **Figure S11.** ^13^C NMR: 3-Methoxy-Piperidine-7-carboxylic acid 2-acetyl-phenyl ester **(3h)**  **Figure S12**. FT-IR: 3-Methoxy-Piperidine-7-carboxylic acid 2-acetyl-phenyl ester **(3h)**  **Figure S13.** ^1^H NMR: Piperidine-7-carboxylic acid 2-acetyl-phenyl ester **(3i)**  **Figure S14.** ^13^C NMR: Piperidine-7-carboxylic acid 2-acetyl-phenyl ester **(3i)**  **Figure S15**. FT-IR: Piperidine-7-carboxylic acid 2-acetyl-phenyl ester **(3i)**  **Figure S16.** ^1^H NMR: *N*-phenylacetamide **(6l)**  **Figure S17.** FT-IR: *N*-phenylacetamide **(6l)**  **Figure S18.** ^1^H NMR: *N*-benzylacetamide **(6m)**  **Figure S19.** FT-IR: *N*-benzylacetamide **(6m)** | **S11-S12**  **S13-S16**  **S17**  **S18**  **S19**  **S20**  **S21**  **S22**  **S23**  **S24**  **S25**  **S26** |

**Figure S1.** Synthesis of enol and phenol carbamates by oxidative coupling.

**Figure S2**. Preparation procedure of Cu@Sal-CS.


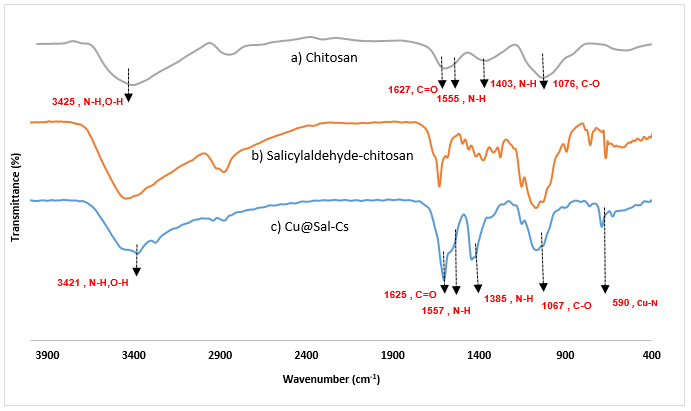


**Figure S3.** FT-IR spectra: **a)** Chitosan, **b)** Salicylaldehyde-chitosan, and **c)** The Cu@modified-chitosan sample.


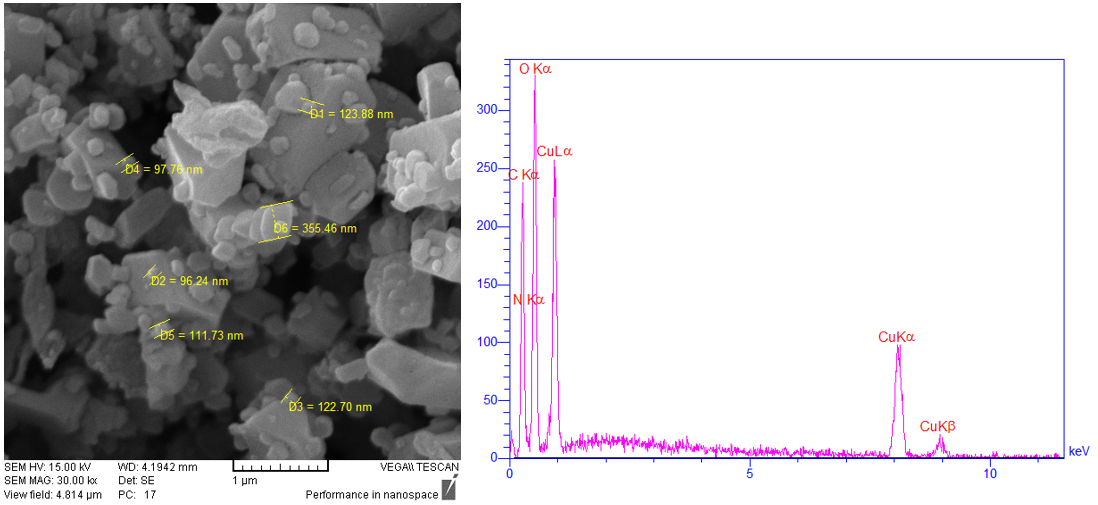


**Figure S4.** Analysis of Cu@modified-chitosan, left: SEM image, right: The EDX diagram.

***
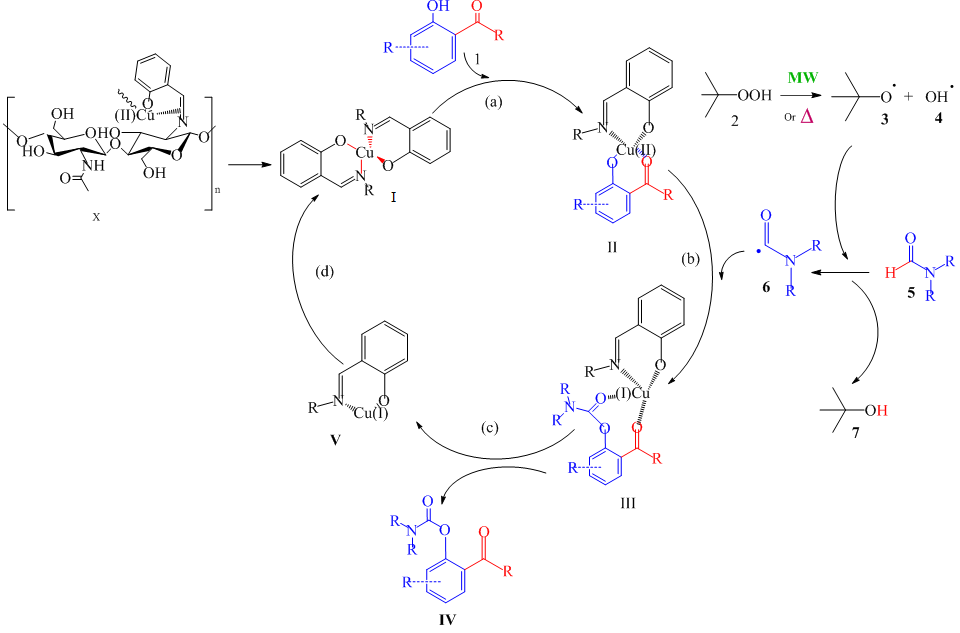
***

**Figure S5**. Proposed mechanism for Cu@Sal-CS catalyzed synthesis of carbamates.

**Figure S6.** Synthesis of amides by oxidative coupling.


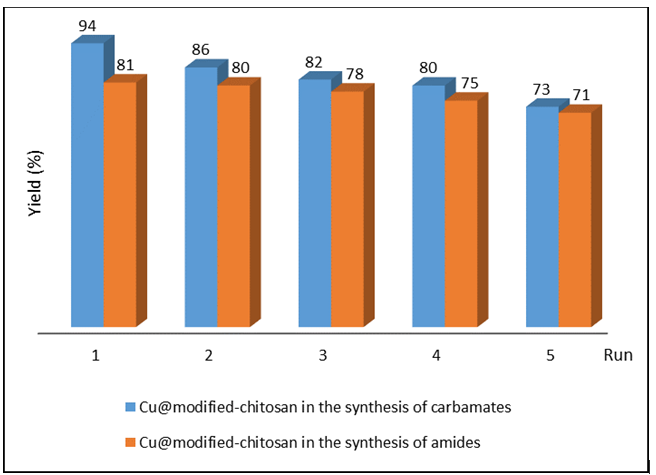


**Figure S7.** Reusability of the catalyst in the model coupling reactions at the optimized condition.


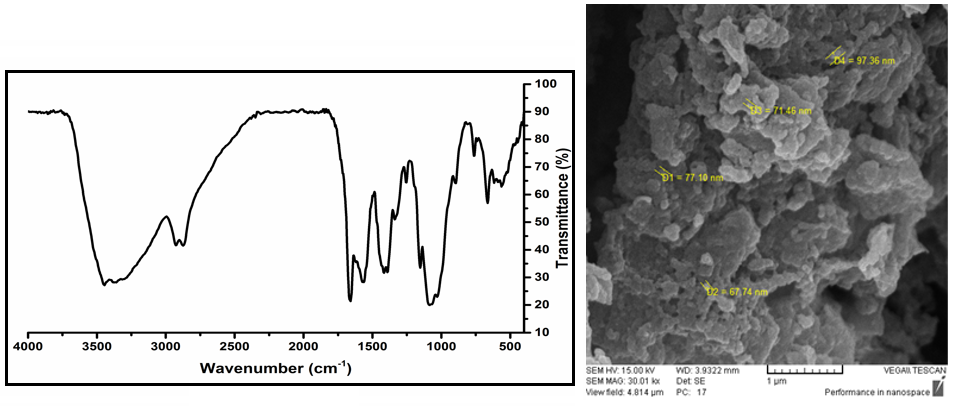


**Figure S8.** Left: FT-IR spectra: Recycled catalyst, right: Scanning electron micrograph of the recycled catalyst.

**Figure S9.** Schematic structure of the synthesized Cu@Sal-CS.

**^1^H NMR, ^13^C NMR and IR spectral data for selected compounds (3h, 3i, 6l, 6m)**

**3-Methoxy-Piperidine-7-carboxylic acid 2-acetyl-phenyl ester (3h)**

Yield: 94 %. Yellow liquid. ^1^H NMR (500 MHz, CDCl_3_): δ (ppm) 7.79(dd, *J_32_*=8.79, *J_31_*=0.57, 1H, Ar-H), 6.8 (dd, *J_23_*=8.78, *J_21_*=2.42, 1H, Ar-H), 6.67 (d, *J*=2.18, 1H, Ar-H), 3.58 (s ,3H, OCH3), 3.54 (s, 2H, Ar-H), 3.51(s, 2H, Ar-H), 2.65(s, 1H, COCH_3_), 1.06(m, 6H, Ar-H). ^13^C NMR (75 MHz, CDCl_3_): δ (ppm): 196.5 (C_7_), 164 (C_8_), 153 (C_6_), 152 (C_4_), 133(C_5_), 124(C_3_), 112 (C_2_), 109 (C_1_), 56 (OMe), 46&45 (C_C, B_), 30 (Me), 26&24 (C_E, F_), 24 (C_D_). FT IR (KBr pellet, cm^−1^):.2937, 2858, 1720, 1674, 1608, 1439, 1357, 1257, 1138, 1068, 1022.

**Piperidine-7-carboxylic acid 2-acetyl-phenyl ester (3i)**

Yield: 74 %. Yellow oil. ^1^H NMR (500 MHz, CDCl_3_): δ (ppm) 7.78 (dd, *J_12_*= 7.79, *J_13_*:1.67, 1H, Ar-H), 7.52 (ddd, *J_34_*= 7.75, *J_32_*= 7.99,7.51, *J_31_*= 1.67, 1H, Ar-H), 7.29 (ddd, *J_21_*=7.59, J_23_=7.57, J_21_=1.08, 1H, Ar-H), 7.16 (dd, *J43*=8.15, J42=1, Ar-H), 3.71 (s, 2H, Ar-H), 3.56 (s, 2H, Ar-H), 2.59 (s, 3H, COCH_3_), 1.72 (m, 6H, Ar-H). ^13^C NMR (75 MHz, CDCl_3_): δ (ppm): 197 (C_4_), 154 (C_8_), 150 (C_5_), 134 (C_3_), 132 (C_6_), 130 (C_1_), 126 (C_2_), 124 (C_4_), 46, 45.7 (C_B_ and C _C_), 31 (Me), 25 (C_E, F_), 24 (C_D_). FT IR (KBr pellet, cm^-1^): 2923, 2856, 1722, 1697, 1598, 1423, 1269, 1201, 1135, 1018, 960, 754, 595.

***N*-phenylacetamide (6l)**

Yield: 85 %. White solid. Mp: 111-113 ^o^C. ^1^H NMR (500 MHz, DMSO-*d6*): δ (ppm) 9.92 (s, 1H), 7.57 (d, *J* = 7.5 Hz, 2H), 7.27 (t, *J* = 7.5 Hz, 2H), 7.00 (t, *J* = 7.5 Hz, 1H), 2.03 (s, 3H). FT IR (KBr pellet, cm^-1^): 3293, 2924, 2855, 1662, 1603, 1549, 1495, 1433, 1317, 1259, 753.

***N*-benzylacetamide (6m)**

Yield: 92%. White solid. Mp: 59-61 ^o^C. ^1^H NMR (400 MHz, CDCl_3_): δ (ppm) 7.30-7.37 (m, 5H), 5.83 (br s, 1H), 4.45 (s, 2H), 2.05 (s, 3H). FT IR (KBr pellet, cm^-1^) 3291, 3084, 2926, 2855, 1641, 1550, 1442, 1366, 1287, 1080, 1025, 742, 693.

**IR spectral data for some compounds**

**2-formylphenyl dimethylcarbamate (3a)**

Yield: 94 %. Colorless liquid. FT IR (KBr pellet, cm^-1^): 2937, 2856, 1724, 1699, 1450, 1425, 1259, 1232, 1209, 1186, 1139, 1020, 852, 756.

**(4-fluorophenyl)(piperidin-1-yl)methanone (6a)**

Yield.: 83%. White solid. Mp: 167-170 ^o^C.FT IR (KBr pellet, cm^-1^): 3080, 2997, 2896, 2852, 2677, 2594, 2563, 1681, 1604, 1510, 1429, 1294, 1234, 1159, 923, 852, 769, 682, 611, 547

**4-bromo-2-formylphenyl dimethylcarbamate (3b)**

Yield: 89 %. White solid: Mp: 62-65 ^o^C. FT IR (KBr pellet, cm^-1^): 2954, 2923, 2854, 1728, 1685, 1643, 1589, 1477, 1388, 1271, 1224, 1182, 1122, 1078, 979, 881, 813, 727.

**(4-fluorophenyl)(morpholino)methanone (6c)**

Yield: 86%. Colorless liquid. FT IR (KBr pellet, cm^-1^): 3068, 2967, 2923, 2854, 1730, 1699, 1635, 1600, 1506, 1456, 1431, 1272, 1232, 1153, 1112, 1016, 848, 761.

**(4-chlorophenyl)(piperidin-1-yl)methanone (6d)**

Yield: 75%. Colorless liquid. FT IR (KBr pellet, cm^-1^): 3064, 2958, 2927, 2862, 2825, 2684, 2650, 2557, 2515, 1691, 1591, 1477, 1440, 1409, 1313, 1294, 167, 1170, 1143, 1132, 1047, 914, 746, 709, 646, 561.

**2-nitrophenyl dimethylcarbamate (3e)**

Yield: 93 %. Yellow liquid, FT IR (KBr pellet, cm^-1^): 3028, 2929, 2856, 1735, 1606, 1529, 1475, 1452, 1390, 1353, 1263, 1224, 1157, 1004, 869, 831, 783, 742.

**4-bromo-2-formylphenyl morpholine-4-carboxylate (3k)**

Yield: 95%. White solid. Mp: 131-133 ^o^C. FT IR (KBr pellet, cm^-1^): 2958, 2923, 2854, 1731, 1683, 1471, 1419, 1402, 1242, 1211, 1182, 1118, 1058, 970, 806, 746, 628, 459.

**2-acetylphenyl morpholine-4-carboxylate (3n)**

Yield: 66 %. Yellow oil. FT IR (KBr pellet, cm^-1^): 2954, 293, 2854, 1733, 1691, 1602, 1454, 1421, 1361, 1244, 1207, 1118, 1056, 958, 854, 754, 574.

**2-nitrophenyl morpholine-4-carboxylate (3o)**

Yield: 69%. White solid. Mp: 80-84 ^o^C. FT IR (KBr pellet, cm^-1^): 2952, 2923, 2854, 1782, 1724, 685, 1662, 1606, 1529, 1460, 1392, 1352, 1276, 1244, 1164, 1076, 972, 856, 748.

**2-formyl-6-methoxyphenyl dimethylcarbamate (3r)**

Yield: 92%. White solid. Mp: 69-71 ^o^C. FT IR (KBr pellet, cm^-1^): 2941, 2844, 1728, 1699, 1585, 1483, 1440, 1384, 1319, 1272, 1207, 1159, 1064, 1004, 950.

**2-formyl-6-methoxyphenyl morpholine-4-carboxylate (3s)**

Yield: 85 %. Colorless liquid. FT IR (KBr pellet, cm^-1^): 3440, 3388, 2958, 2925, 2854, 1731, 1579, 1429, 251, 1114, 1039, 831.

***Tert*-butyl (Z)-3-((dimethylcarbamoyl) oxy) but-2-enoate (3t)**

Yield: 95 %. Yellow liquid, FT IR (KBr pellet, cm^-1^): 2977, 2933, 2360, 2325, 1726, 1668, 1502, 1452, 1396, 1373, 1321, 1228, 1164, 1132, 1043, 1022, 904, 750.

**Allyl (*Z*)-3-((dimethylcarbamoyl) oxy) but-2-enoate (3u)**

Yield: 91 %. Yellow oil. FT IR (KBr pellet, cm^-1^ ): 2941, 1726, 1650, 1506, 1396, 1271, 1211, 1134, 1026, 823, 748.

**3-oxocyclohex-1-en-1-yl dimethylcarbamate (3w)**

Yield: 99 %. Yellow oil. FT IR (KBr pellet, cm^-1^): 2952, 2925, 2854, 1733, 1670, 1488, 1458, 1363, 1159, 1130, 964, 879.

**5, 5-dimethyl-3-oxocyclohex-1-en-1-yl dimethylcarbamate (3x)**

Yield: 99%. White solid. Mp: 87-90 ^o^C. FT IR (KBr pellet, cm^-1^): 2925, 2862, 1720, 1658, 1645, 1618, 1581, 1454, 1386, 1257, 1163, 1076, 960, 902, 865, 748.


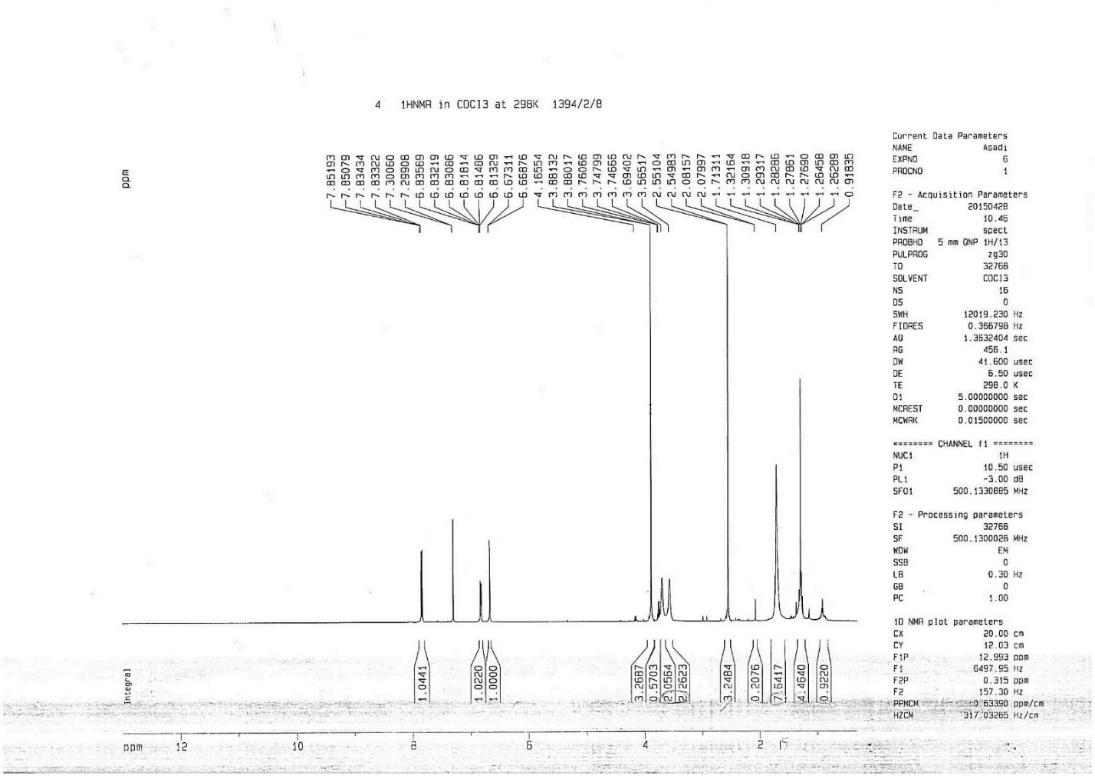


**Figure S10.** ^1^H NMR: 3-Methoxy-Piperidine-7-carboxylic acid 2-acetyl-phenyl ester **(3h)**


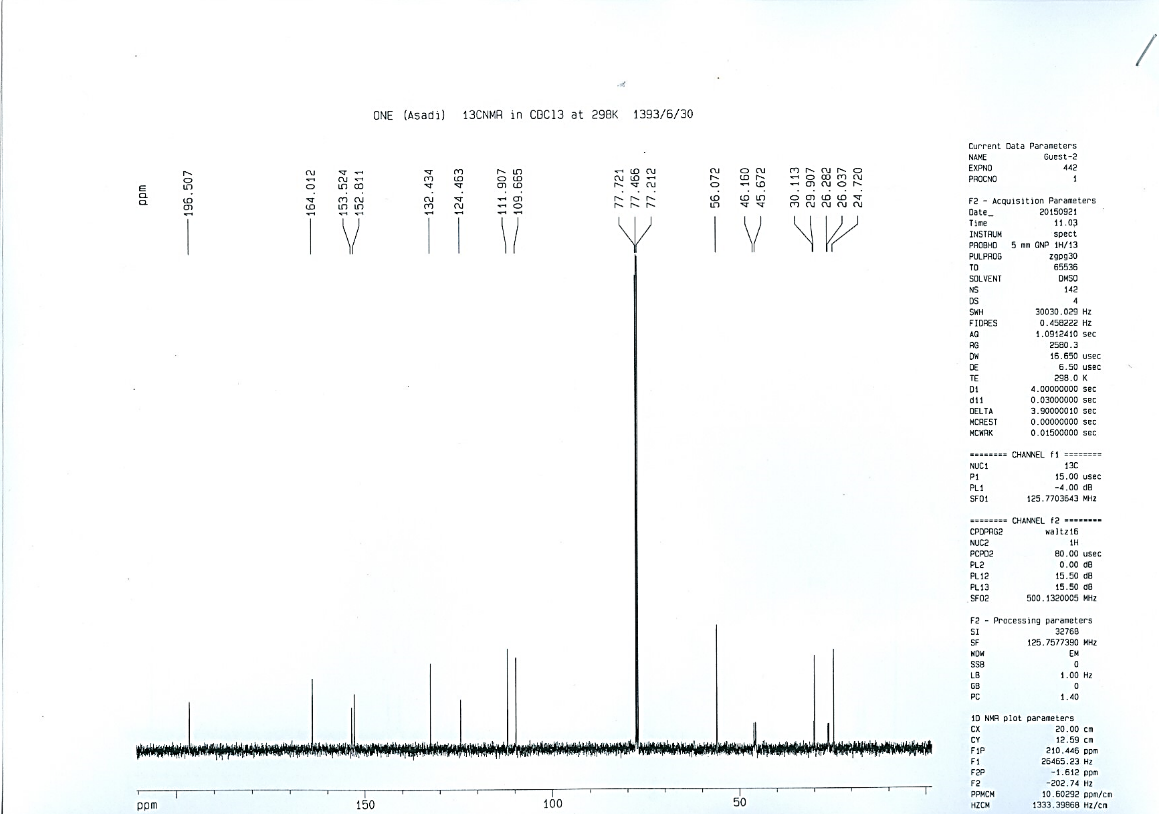


**Figure S11.** ^13^C NMR: 3-Methoxy-Piperidine-7-carboxylic acid 2-acetyl-phenyl ester **(3h)**


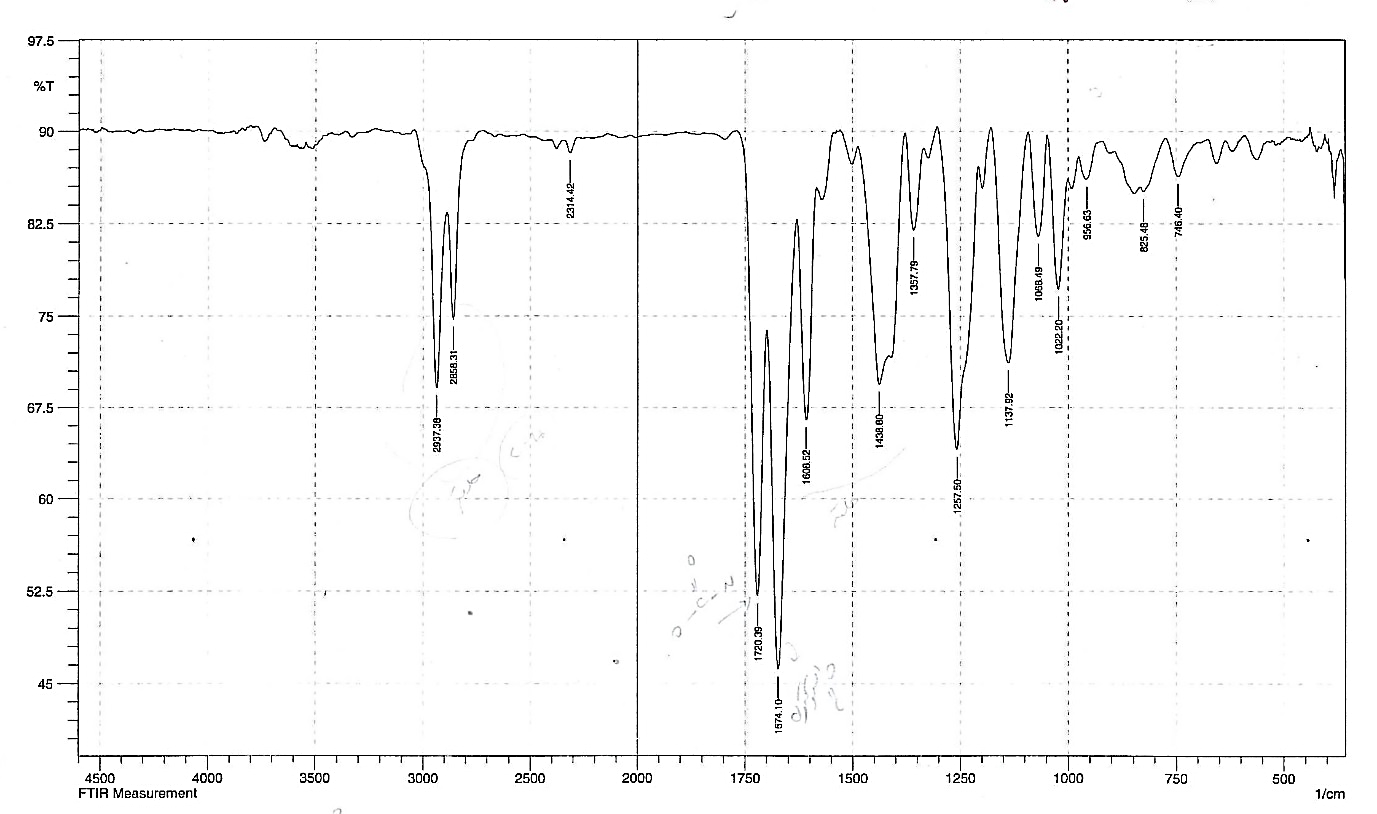


**Figure S12**. FT-IR: 3-Methoxy-Piperidine-7-carboxylic acid 2-acetyl-phenyl ester **(3h)**

**
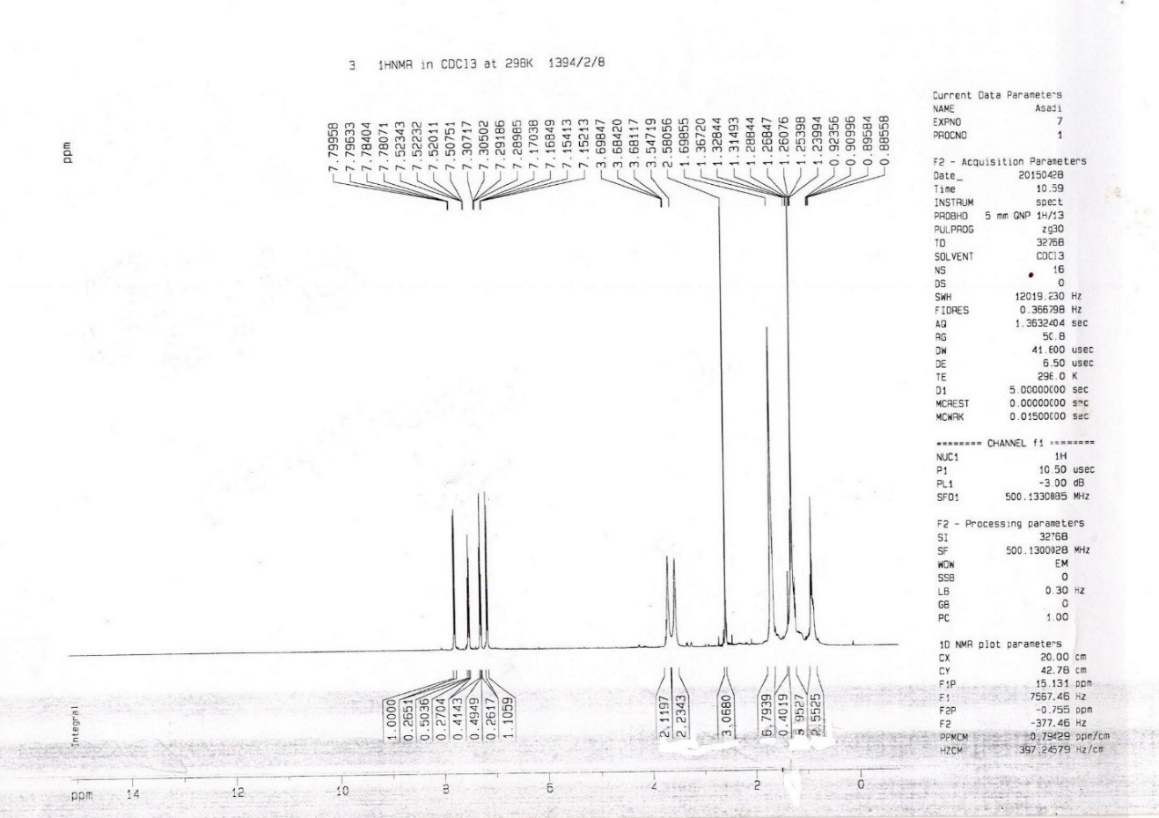
**

**Figure S13.** ^1^H NMR: Piperidine-7-carboxylic acid 2-acetyl-phenyl ester **(3i)**


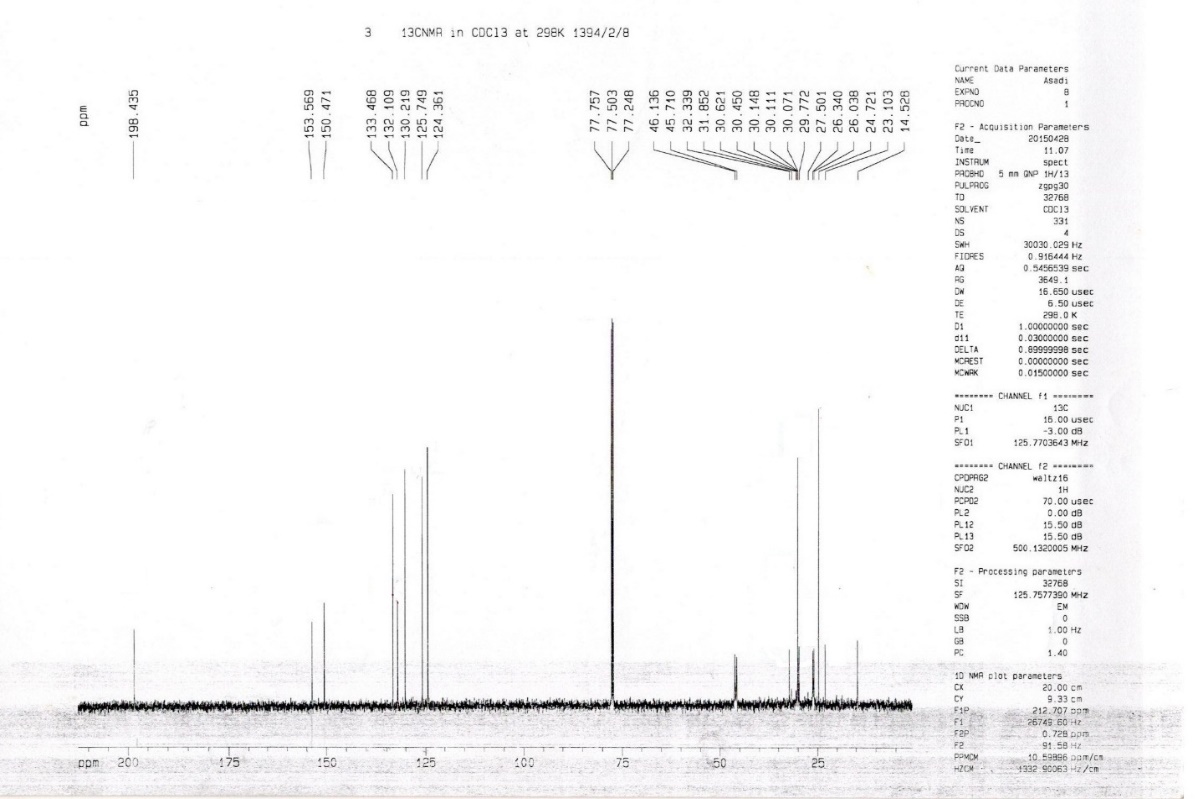


**Figure S14.** ^13^C NMR: Piperidine-7-carboxylic acid 2-acetyl-phenyl ester **(3i)**


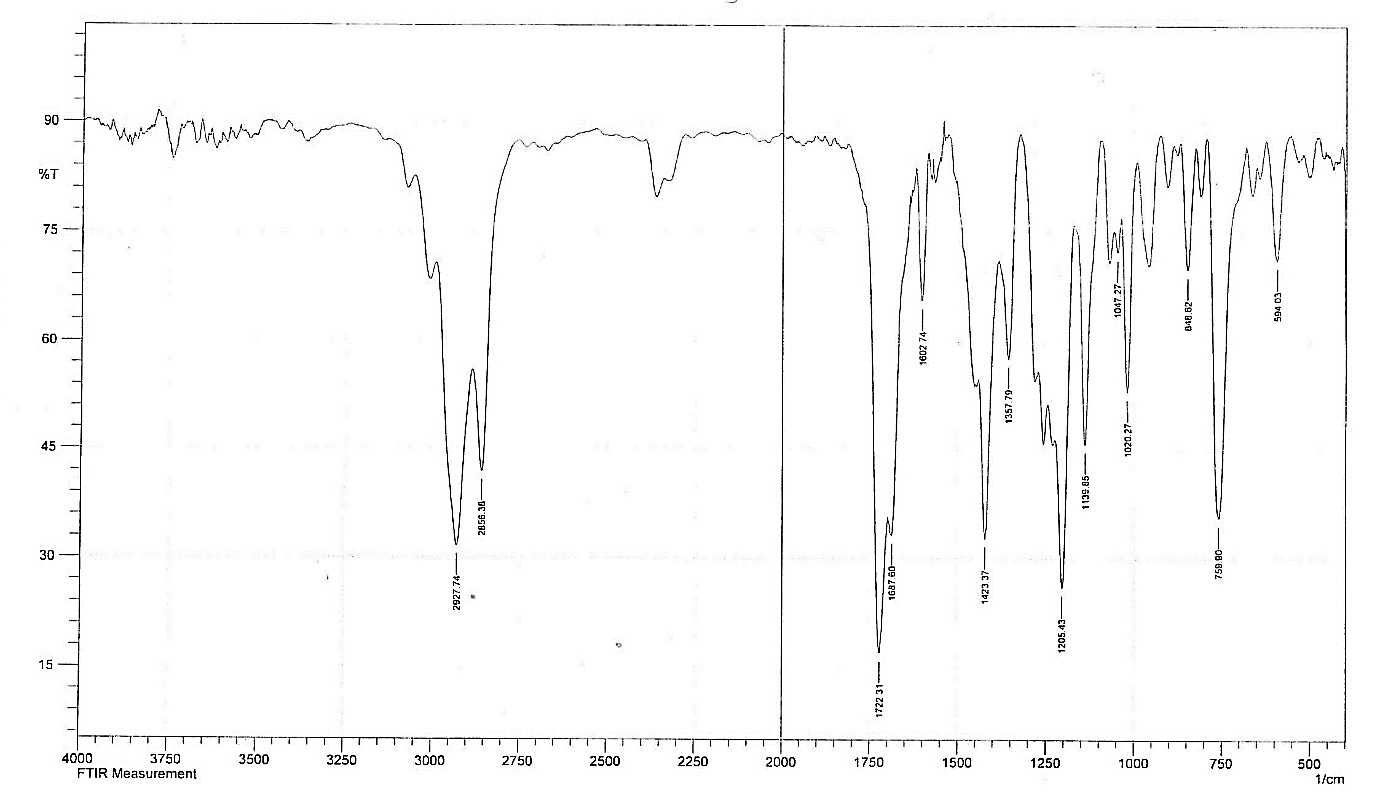


**Figure S15**. FT-IR: Piperidine-7-carboxylic acid 2-acetyl-phenyl ester **(3i)**


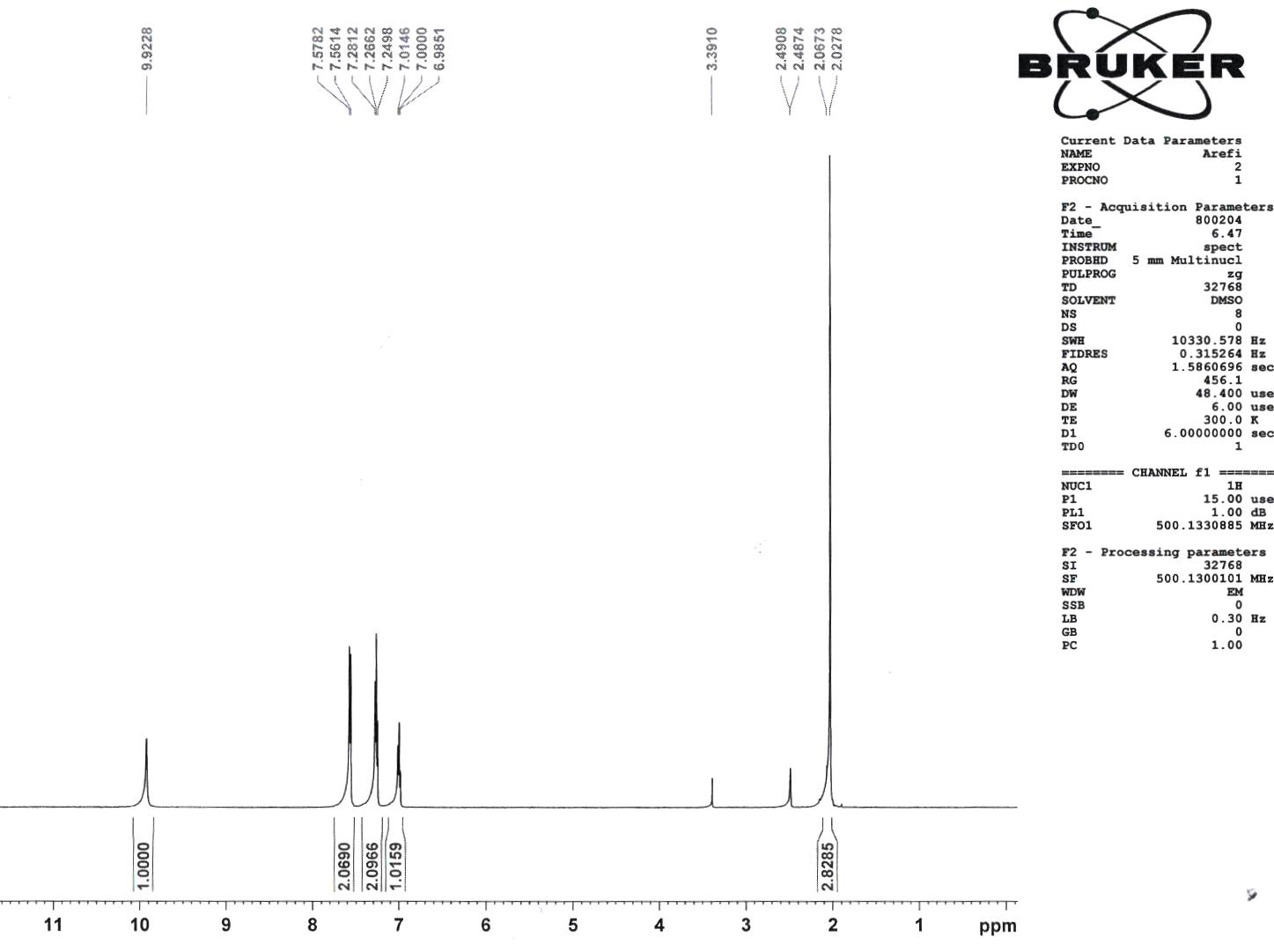


**Figure S16.** ^1^H NMR: *N*-phenylacetamide **(6l)**


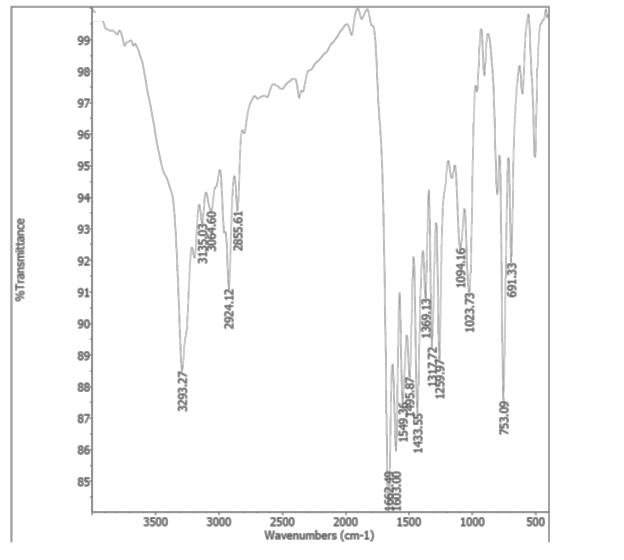


**Figure S17.** FT-IR: *N*-phenylacetamide **(6l)**


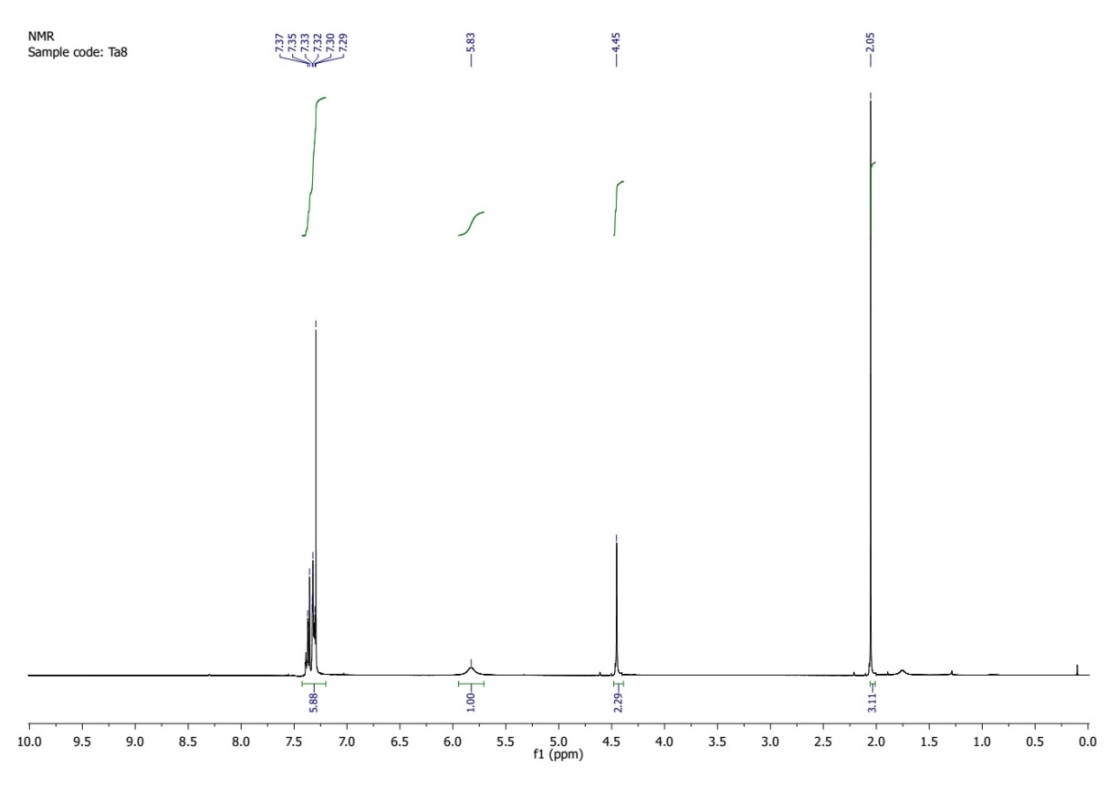


**Figure S18.** ^1^H NMR: *N*-benzylacetamide **(6m)**

**Figure S19.** FT-IR: *N*-benzylacetamide **(6m)**
